# Metabolic Profiling of Resistant and Susceptible Tobaccos Response Incited by *Ralstonia pseudosolanacearum* Causing Bacterial Wilt

**DOI:** 10.3389/fpls.2021.780429

**Published:** 2022-01-07

**Authors:** Liang Yang, Zhouling Wei, Marc Valls, Wei Ding

**Affiliations:** ^1^Laboratory of Natural Products Pesticides, College of Plant Protection, Southwest University, Chongqing, China; ^2^Centre for Research in Agricultural Genomics (CRAG), CSIC-IRTA-UAB-UB, Campus UAB, Barcelona, Spain; ^3^Genetics Section, Facultat de Biologia, Universitat de Barcelona, Barcelona, Spain

**Keywords:** metabolomics, GC-MS, tobacco, *Ralstonia pseudosolanacearum*, amino acid

## Abstract

The causal agent of bacterial wilt, *Ralstonia pseudosolanacearum*, can cause significant economic losses during tobacco production. Metabolic analyses are a useful tool for the comprehensive identification of plant defense response metabolites. In this study, a gas chromatography-mass spectrometry (GC-MS) approach was used to identify metabolites differences in tobacco xylem sap in response to *R. pseudosolanacearum* CQPS-1 in two tobacco cultivars: Yunyan87 (susceptible to *R. pseudosolanacearum*) and K326 (quantitatively resistant). Metabolite profiling 7 days post inoculation with *R. pseudosolanacearum* identified 88 known compounds, 42 of them enriched and 6 depleted in the susceptible cultivar Yunyan87, while almost no changes occurred in quantitatively resistant cultivar K326. Putrescine was the most enriched compound (12-fold) in infected susceptible tobacco xylem, followed by methyl-alpha-d-glucopyranoside (9-fold) and arabinitol (6-fold). Other sugars, amino acids, and organic acids were also enriched upon infection. Collectively, these metabolites can promote *R. pseudosolanacearum* growth, as shown by the increased growth of bacterial cultures supplemented with xylem sap from infected tobacco plants. Comparison with previous metabolic data showed that beta-alanine, phenylalanine, and leucine were enriched during bacterial wilt in both tobacco and tomato xylem.

## Introduction

Tobacco (*Nicotiana tabacum* L.) is the most important non-edible agricultural product worldwide (Reichert et al., 2019; Mirkarimi et al., 2021). China is the largest tobacco producer worldwide, growing 1,100,000 ha by almost 1,520,000 farmers that yield 2,610,000 tons of dried leaves every year (Shahbandeh, 2021). Bacterial wilt, a disease caused by the bacterium *Ralstonia solanacearum* species complex, affects tobacco production in numerous countries (Mansfield et al., 2012; Prior et al., 2016; Jiang et al., 2017). In the field, pathogen-infected tobacco exhibits partial wilting symptoms and premature yellowing of leaves, and one side of the stem develops a brown discoloration. High disease incidence results in production losses, leading to serious damage to the tobacco industry. Bacterial wilt is currently widespread in the main tobacco production regions of Yunnan, Sichuan, Guizhou, Guangxi, Hunan, Hubei, Chongqing, and Guangdong provinces in China (Liu et al., 2017b).

Due to the bacterial aggressiveness, large host range, and broad geographical distribution, control of bacterial wilt has been challenging (Genin and Denny, 2012). Understanding the molecular mechanisms mediating interactions between pathogen and plants is fundamental to develop effective management strategies for disease control. Genomics, transcriptomics, proteomics, and metabolomics are currently applied to study the interaction between crops and pathogens (Planas-Marques et al., 2018; Gao et al., 2019; Gong et al., 2020; de Pedro-Jove et al., 2021). The complete genomic sequence of several *R. pseudosolanacearum* strains infecting tobacco in China have been recently published (Liu et al., 2017a). Global transcriptional gene expression of tomato and tobacco in response to *R. pseudosolanacearum* infection has been analyzed using comparative transcriptome analysis, showing the enrichment in two groups of gene ontology terms regarding glutathione and flavonoid metabolisms in resistant tobacco cultivars (Gao et al., 2019). Proteomic and transcriptomic results demonstrated that gamma-aminobutyric acid (GABA) biosynthesis pathway and methionine cycle (MTC) play a key role in pathogenic interaction between *R. solanacearum* and tomato plants (Wang et al., 2019). However, metabolite changes in tobacco after infection of *R. pseudosolanacearum* have not been addressed up to now.

Metabolomics is playing an important role in identification of the key metabolites in plant adaptation (Zhang et al., 2018; Lowe-Power et al., 2018b; Zeiss et al., 2019). GC-MS metabolic profiling has been widely used to detect metabolite changes in various tobacco cellular processes (Zhang et al., 2018; Tsaballa et al., 2020). Recent studies have investigated the metabolites changes of tobacco leaves from different geographical origins in China and demonstrated the key metabolic pathways related the environmental adaption (Liu et al., 2020). Certain metabolites such as phenolic amino acids, phenylpropanoids, linoleic acid, and hydroxycinnamic acid amides are changed after *Phytophthora parasitica* var. nicotianae inoculation in tobacco plants (Cho et al., 2012). This same approach applied to tomato plants challenged with *R. solanacearum* identified several enriched metabolites in the xylem sap, some of which can be carbon or nitrogen sources for *R. solanacearum* growth (Lowe-Power et al., 2018a). Glutamine and asparagine were identified as primary resources consumed by *R. solanacearum* during its colonization phase (Gerlin et al., 2021). Flavonoids and hydroxycinnamic acids are also of prime importance in the tomato defense response to *R. solanacearum* invasion (Lowe-Power et al., 2015; Zeiss et al., 2019). However, the metabolic profiles of different tobacco cultivars in response to *R. pseudosolanacearum* infection remain largely unknown.

In this work, metabolic profiling using GC-MS was performed to investigate the metabolites changes responses to *R. pseudosolanacearum* infection in two different tobacco cultivars (Yunyan87 and K326). On the basis of the functions of the identified metabolites, we studied the metabolic significance concerning the susceptibility of tobacco to *R. pseudosolanacearum*.

## Materials and Methods

### Plant Growth Conditions and Bacterial Strain

Two tobacco cultivars (bacterial wilt-susceptible cv. Yunyan 87 and quantitatively resistant breeding line K326) were used in this experiment (Cai et al., 2021). Tobacco seeds were sown in plant growing mix soil in a 28°C climate chamber with a light/dark cycle of 14 h/10 h. Tobacco seedlings were transplanted 28 days post sowing into individual 12 cm pots containing mix soil. After 10 days post transplanted, tobacco plants were used for infection assay.

The highly aggressive *R. pseudosolanacearum* strain CQPS-1 (phylotype I, race 1, biovar 3) isolated from tobacco stems in China (Liu et al., 2017a) was used. The bacterial strain was grown in complete BG liquid medium supplemented with 0.5% glucose at 30°C and stored at −80°C in nutrient broth with 25% glycerol. Boucher’s minimal medium (MM) pH 7.0 containing 20 mM glucose, 0.5 g/L (NH_4_)_2_SO_4_, 3.4 g/L KH_2_PO_4_, 0.125 mg/L FeSO_4_·7H_2_O, and 62.3 mg/L MgSO_4_ was used for experiments.

### Virulence Assay

Soil-drenching assays were performed to investigate the symptomatology of two different tobacco cultivars (Yunyan 87 and K326) after infection. To this end, 6-week-old tobacco plants were soil-soak inoculated by drenching with *R. pseudosolanacearum* bacterial suspension (1 × 10^8^ colony forming units-CFU−/ml). Infected tobacco plants were moved into a growth chamber at 28°C with a 14/10 h light/dark cycle. Bacterial wilt symptoms were scored daily using a disease index scale from 0 to 4 (0 indicates no symptoms; 1: 1–25% of leaves wilted; 2: 26–50% of leaves wilted; 3: 51–75% of leaves wilted; 4: 76–100% of leaves wilted). Individual treatments contained 16 plants for each independent experiment, and the assay was repeated three times. The disease index was calculated as a weighted average.

### Bacterial Density in Two Tobacco Cultivars

Bacterial populations were determined by harvesting 100 mg tobacco stem as previously described (Yang et al., 2018). Samples were disinfected and transferred into a 2.5-ml sterile centrifuge tube and ground with sterile glass beads using the MP Biomedicals FastPrep. Next, serially diluted homogenates were plated on SMSA medium, and colonies were counted after 2 days incubation at 28°C (Elphinstone et al., 1996). Each treatment contained 8 samples. The assay was repeated twice.

### Collection of Xylem Saps From Tobacco Stems

For the untargeted metabolomics experiments, tobacco plants were used 7 days after soil-soak inoculation with *R. pseudosolanacearum* to collect xylem sap as described previously with minor modifications (Siebrecht et al., 2003). Samples from healthy and infected tobacco stem tissues were harvested by centrifugation at 4,000 × *g* for 5 min at 4°C, and the supernatants were transferred into prechilled tubes. Samples were then sterilized with 0.22-μM filters and stored at −80°C until analysis. Each sample contains xylem sap from six tobacco plants.

### Bacterial Growth Supplemented With Xylem Sap From Tobacco Cultivars

Xylem sap was harvested as described above. Xylem sap was collected from at least six healthy or infected tobacco plants and sterilized through a 0.22-μm filter. An overnight bacterial culture was washed and inoculated in MM, and 50 μl bacteria suspension were mixed with equal volume of xylem sap from healthy/infected plants and then transferred to a 96-well microplate. Fresh MM (50 μl) was added to control wells. The plate was returned into plate reader, and the bacterial OD600 was measured every 4 h during 40 h of cultivation. The experiment was repeated twice.

### GC-MS Metabolomics Analysis

GC-MS analysis was performed for metabolite profiling by a GC-MS system (Agilent 7890A/5975C; Dunn et al., 2011). Briefly, 100 μl xylem sap was transferred into a 1.5-ml centrifuge tube with 400 μl of prechilled methanol and mixed for 60 s. Nonadecylic acid and d4-alanine were used as internal quantitative standard. After centrifugation for 10 min, supernatant was transferred into new tube. 60 μl methoxyamine pyridine solution and BSTFA reagent were added to the mixture and left to react for 90 min at 37°C. GC-MS was performed on a HP-5MS capillary column (Agilent J & w Scientific, Folsom, CA, United States), under a constant flow of 1 ml·min^−1^ helium. Samples were injected in split mode in a 20:1 split ratio by the auto-sampler. Column temperature was set at 60°C for 2 min, then ramped to 300°C by 10°C min^−1^, and held constant for 5 min. Ions were generated with a − 70 eV and 1800 V ionization energy, and masses (35–750 *m/z*) were acquired. Peak detection and deconvolution was performed in the Serveis Científicotècnics of the University of Barcelona. Metabolites were relatively quantified by peak area for the quantification ion.

### Metabolomics Data Analysis

The GC-MS datasets were analyzed with XCMS online software (https://xcmsonline.scripps.edu/; Forsberg et al., 2018). The raw GC-MS data were processed with XCMS 3.5 software using an automated cloud-based method to process raw metabolomics data, generating a list of statistically significant features that could be used for biological interpretation. To extract potential differentially concentrated metabolites, the cutoff of *q*-value <0.01 and |fold change| > 2 was applied.

### Statistical Analyses

The data were analyzed by SPSS software using Student’s *t* test under significance levels of 0.05 and 0.01.

## Results

### Phenotypic Observation After *R. pseudosolanacearum* Inoculation

**Figure 1:**
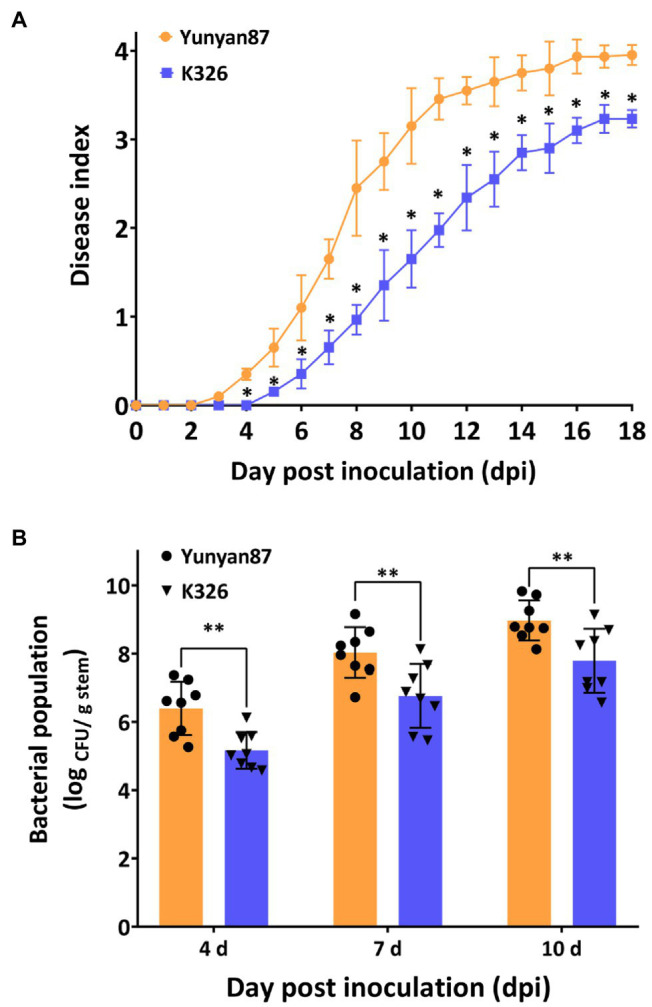
Differential responses of tobacco cultivars Yunyan87 and K326 to *R. pseudosolanacearum* inoculation. **(A)** Bacterial wilt disease index of susceptible cultivar Yunyan87 and quantitatively resistant line K326 scored over time after soil-soak inoculation using a disease index scale from 0 to 4. **(B)** *R. pseudosolanacearum* bacterial populations in stem tissues of the two tobacco cultivars soil-soak inoculated with *R. pseudosolanacearum*. Each bar represents the mean ± standard error of eight experimental replicas. Asterisks indicate significant differences between tobacco cultivar Yunyan87 and K326 according to Student’s *t* test (* indicates *p* < 0.05, ** indicates *p* < 0.01).

Tobacco plants of cultivar Yunyan87 exhibited bacterial wilt on the leaf as early as 3 days post inoculation (dpi). On the contrary, the symptoms on the quantitatively resistant cultivar K326 started to appear at 5 dpi (Figure 1A). 7 days and 10 days after inoculation, the disease index of K326 was 0.65 and 1.65, which were significantly lower than disease index of Yunyan87 with 1.65 and 3.15 (Figure 1A). In agreement with the wilting symptoms, the bacterial population of R. pseudosolanacearum was significantly lower in K326 than in Yunyan87 both at 4, 7, and 10 days post inoculation (Figure 1B).

### *R. pseudosolanacearum* Infection Significantly Alters the Metabolites in Xylem Sap of Tobacco Cultivars

**Figure 2:**
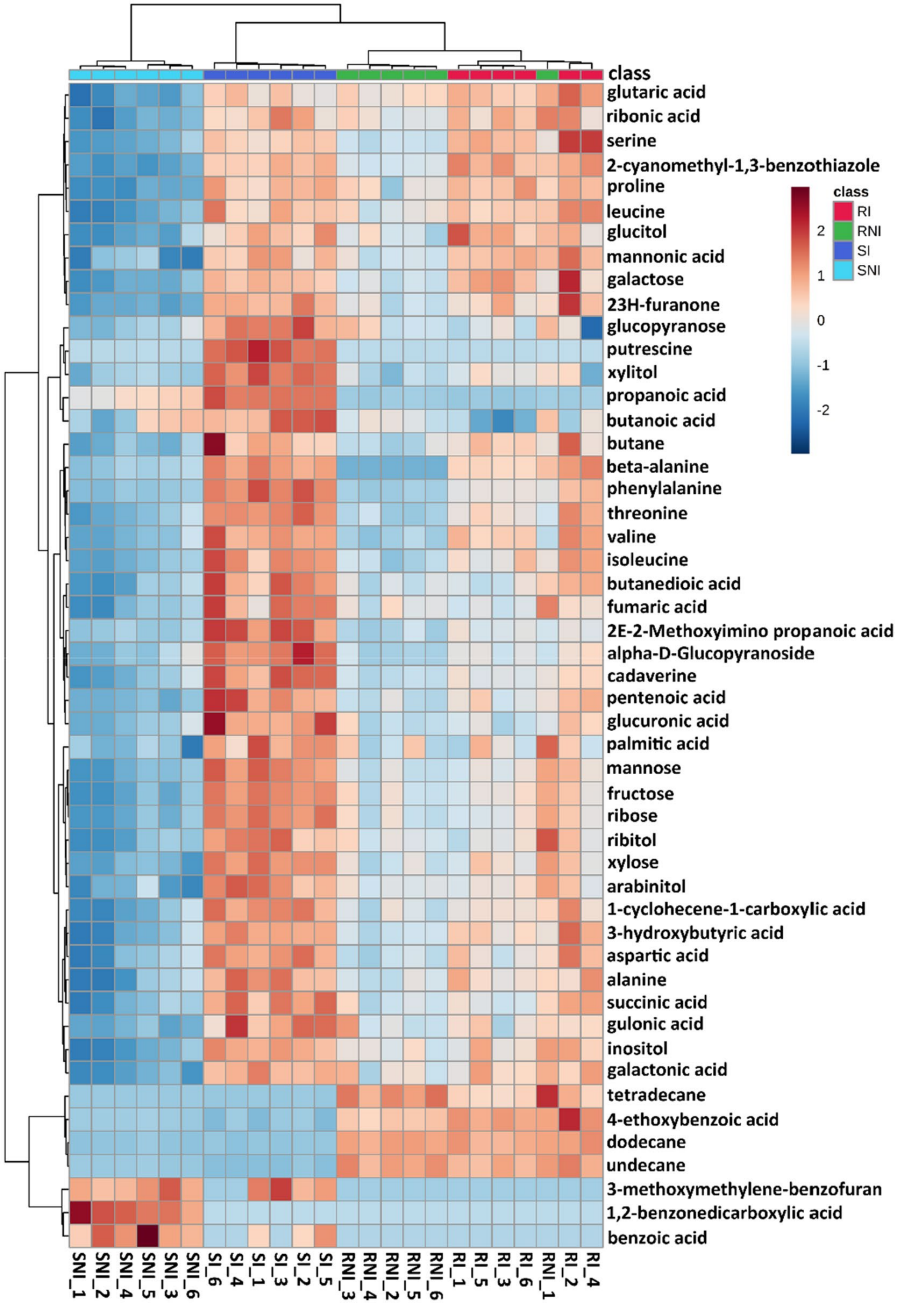
Metabolome of tobacco (susceptible cultivar Yunyan87 and moderately resistant cultivar K326) in response to *R. pseudosolanacearum* infection. Heatmap of differentially expressed metabolites in tobacco cultivars (Yunyan87 and K326) in response to *R. pseudosolanacearum* invasion. Columns correspond to experimental replicas.

The metabolite profiles of xylem sap of tobacco cultivars Yunyan87 and K326 were determined by untargeted metabolome analysis at 7 days post *R. pseudosolanacearum* inoculation (Supplementary Figure S1). The results displayed that *R. pseudosolanacearum* infection changes the chemical composition of tobacco xylem sap (Figure 2). Principal component analysis (PCA) of all samples indicated that the susceptible tobacco cultivar Yunyan87 showed dramatic metabolite changes after *R. pseudosolanacearum* infection, while the quantitatively resistant variety K326 showed few metabolite differences (Supplementary Figure S1). GC-MS analysis of the xylem sap samples detected 88 known compounds, including 48 metabolites identified as differentially concentrated (Figure 2). Interestingly, samples from the inoculated or non-inoculated K326 resistant variety showed few metabolite differences, sometimes hampering their differentiation by clustering (Figure 2).

### Bacterial Wilt Infection Enriches Tobacco Xylem Sap to Favor *R. pseudosolanacearum* Growth

**Figure 3:**
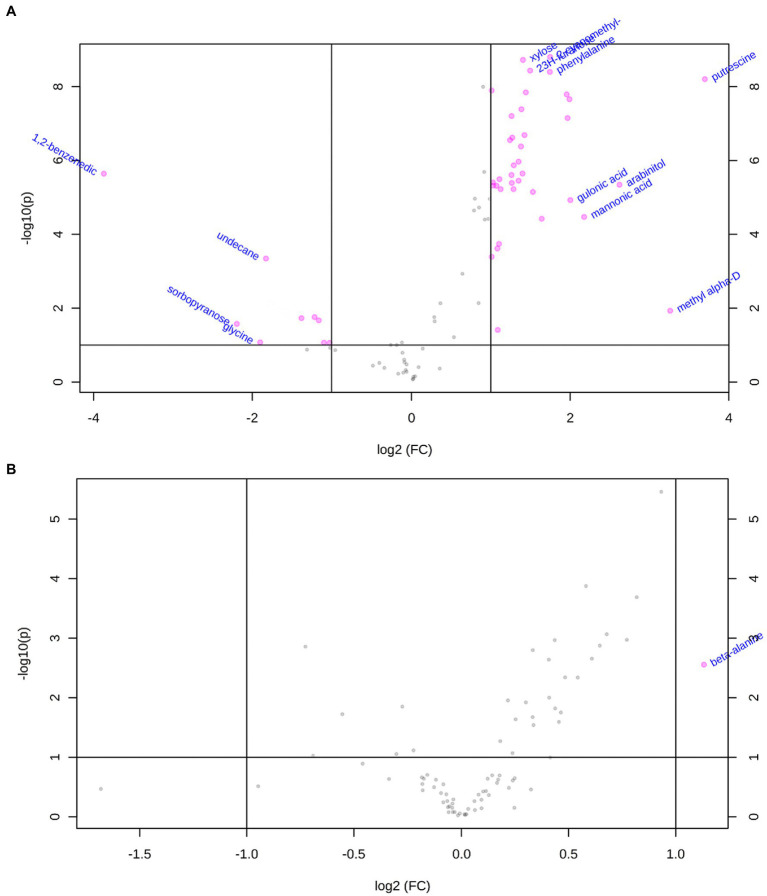
Change of metabolome profile of tobacco in response to *R. pseudosolanacearum* infection. **(A)** Volcano plot showing differentially expressed (DE) metabolites and their value of *p* (p) in susceptible tobacco in response to *R. pseudosolanacearum* invasion. **(B)** Volcano plot showing differentially expressed (DE) metabolites and their value of *p* (p) in resistant tobacco in response to *R. pseudosolanacearum* invasion.

**Figure 4:**
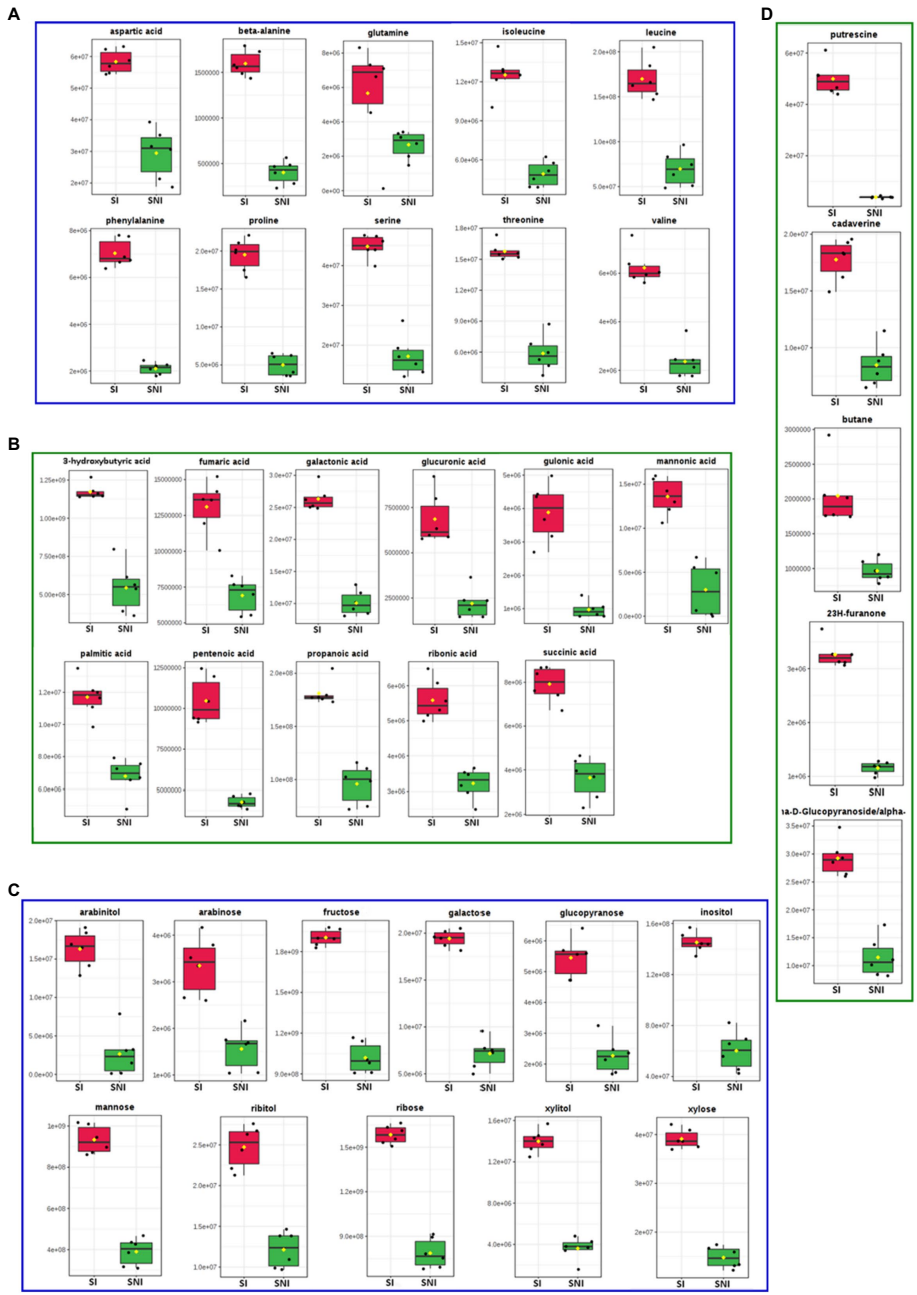
The significantly enriched metabolites of susceptible tobacco cultivar Yunyan87 in response to *R. pseudosolanacearum* infection. **(A)** Enriched amino acids. **(B)** Enhanced organic acids. **(C)** Sugar and polyols compounds enriched. **(D)** Other enriched compounds after *R. pseudosolanacearum* infection. The red indicates the quantification peak area of metabolites from xylem sap of susceptible tobacco cultivar Yunyan87 infected with *R. pseudosolanacearum*, and green indicates quantification peak area of metabolites from xylem sap of healthy tobacco cultivar Yunyan87 plants.

**Figure 5:**
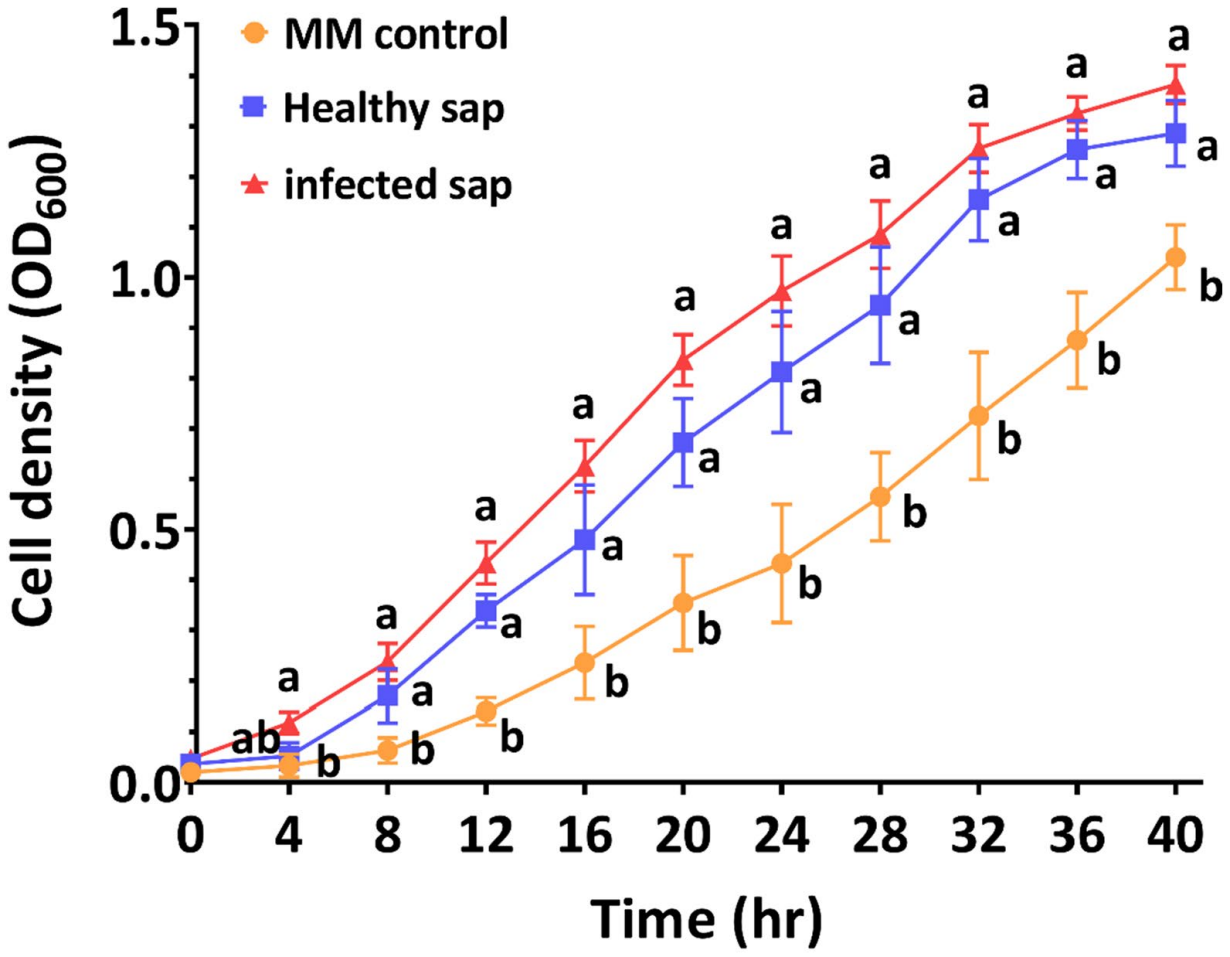
Xylem sap from infected tobacco cultivar Yunyan87 improves *R. pseudosolanacearum* growth *in vitro*. *R. pseudosolanacearum* growth curves in minimal medium or minimal medium supplemented with xylem sap extracted from healthy/infected tobacco. Each bar represents the mean ± SE of three replicas. Different letters indicate xylem sap treatment and control treatment were significantly different (*p* < 0.05).

We focused on the influence of *R. pseudosolanacearum* infection on the metabolites in susceptible tobacco cultivar Yunyan87, whose profile was markedly different in infected compared to healthy plants, as shown by PCA (PC1 = 84.8%) and sample clustering (Supplementary Figures S1, S2). A total of 48 metabolites was identified as differentially abundant, 42 of which were enriched and 6 depleted. The enriched metabolites in xylem sap contained amino acids, organic acids, sugars, and others, suggesting that they could favor *R. pseudosolanacearum* growth *in planta* (Figure 3A, 4). Putrescine was the most enriched compound (12-fold) in infected tobacco xylem, followed by methyl-alpha-d-glucopyranoside (9-fold) and arabinitol (6-fold; Figure 4D, Supplementary Table S2). The enriched amino acids included aspartic acid, beta-alanine, glutamine, isoleucine, leucine, phenylalanine, proline, serine, threonine, and valine (Figure 4A). Among the organic acids were 3-hydroxybutyric acid, fumaric acid, galactonic acid, glucuronic acid, gulonic acid, mannonic acid, palmitic acid, pentenoic acid, propanoic acid, ribonic acid, and succinic acid (Figure 4B). Eleven sugars and polyols were also enriched upon infection: arabinitol, arabinose, fructose, galactose, glucopyranose, inositol, mannose, ribitol, ribose, xylitol, and xylose (Figure 4C). In order to evaluate the effect of xylem sap on *R. pseudosolanacearum* growth, tobacco xylem sap was collected following the same protocol used for metabolic analyses and used to supplement axenic bacterial cultures. As shown in Figure 5, supplementing minimal media with xylem sap significantly improved bacterial growth than mock treatment, and this effect was more apparent although not significantly when sap from *R. pseudosolanacearum*-infected plants was added.

### Moderately Resistant Tobacco Cultivar K326 Displays Few Metabolite Changes in Response to Pathogen Infection

We tested the possibility that xylem sap from moderately resistant tobacco cultivar K326 contained concentrated chemicals that inhibited *R. pseudosolanacearum* growth, but supplementing minimal media with xylem sap from healthy or infected tobacco cultivar K326 improved *R. pseudosolanacearum* growth. As shown in Supplementary Figure S2, the metabolic profile of *R. pseudosolanacearum*-infected xylem sap was similar to that of healthy plants. Only one metabolite (beta-alanine) was identified more abundant in infected plants (Figure 3B). This suggested that sap from *R. pseudosolanacearum*-infected tobacco plants was enriched in nutrients rather than depleted in growth inhibitors.

### *R. pseudosolanacearum* Infection Triggers Different Metabolite Responses in Tobacco and Tomato Plants

To identify key metabolites in the interaction between *R. pseudosolanacearum* and different plant hosts, we compared the metabolites changes of tobacco plant response to *R. pseudosolanacearum* infection to previously published metabolomic data on tomato (Lowe-Power et al., 2018a; Supplementary Table S2). Putrescine was the most enriched compound in xylem sap from both tobacco and tomato plants affected by bacterial wilt (12.97-fold and 75.68-fold, respectively). The concentration of 3-hydroxybutyric acid, galactonic acid, and amino acids beta-alanine, phenylalanine, leucine, and glycine was all enhanced in tobacco and tomato saps infected by *R. pseudosolanacearum* (Supplementary Table S2). However, additional amino acids increased in infected tobacco xylem, including proline, threonine, valine, serine, isoleucine, and glutamine. In turn, sugars and polyols were more enriched in tobacco than in tomato. In particular, methyl-alpha-d-glucopyranoside and arabinitol are major components of tobacco xylem metabolome, but they have not been found in tomato.

## Discussion

### Metabolic Analysis of Two Tobacco Cultivars in Response to Infection by *R. pseudosolanacearum*

Metabolomics is a useful tool for investigation of plant adaptation to pathogen infection (Lowe-Power et al., 2018a). Recently, metabolite profiles of tobacco in response to different pathogens infection have been widely investigated (Lowe-Power et al., 2016; Wang et al., 2020; Zhang et al., 2020). In this study, a total of 88 known compounds were identified in two tobacco cultivars differing for resistance to bacterial wilt (moderately resistant cultivar K326 and susceptible cultivar Yunyan87). Seven days after inoculation, a higher number of differentially concentrated metabolites were identified in Yunyan87 (Figure 2), which could be due to the higher bacterial growth in this susceptible plant (Figure 1B). A recent study also proved that a larger number of enriched metabolites were secreted by susceptible tobacco cultivar Xinhuangjin 1,025 than by resistant Gexin 3 against tobacco black shank disease (Zhang et al., 2020). Unlike the extraction method of xylem sap from tomato plant infected by *R. solanacearum* through root pressure (Lowe-Power et al., 2018a), collection of xylem sap from tobacco stem tissue by centrifugation may have caused some damage to the tissue, leading to leaking of cellular metabolites.

**Figure 6:**
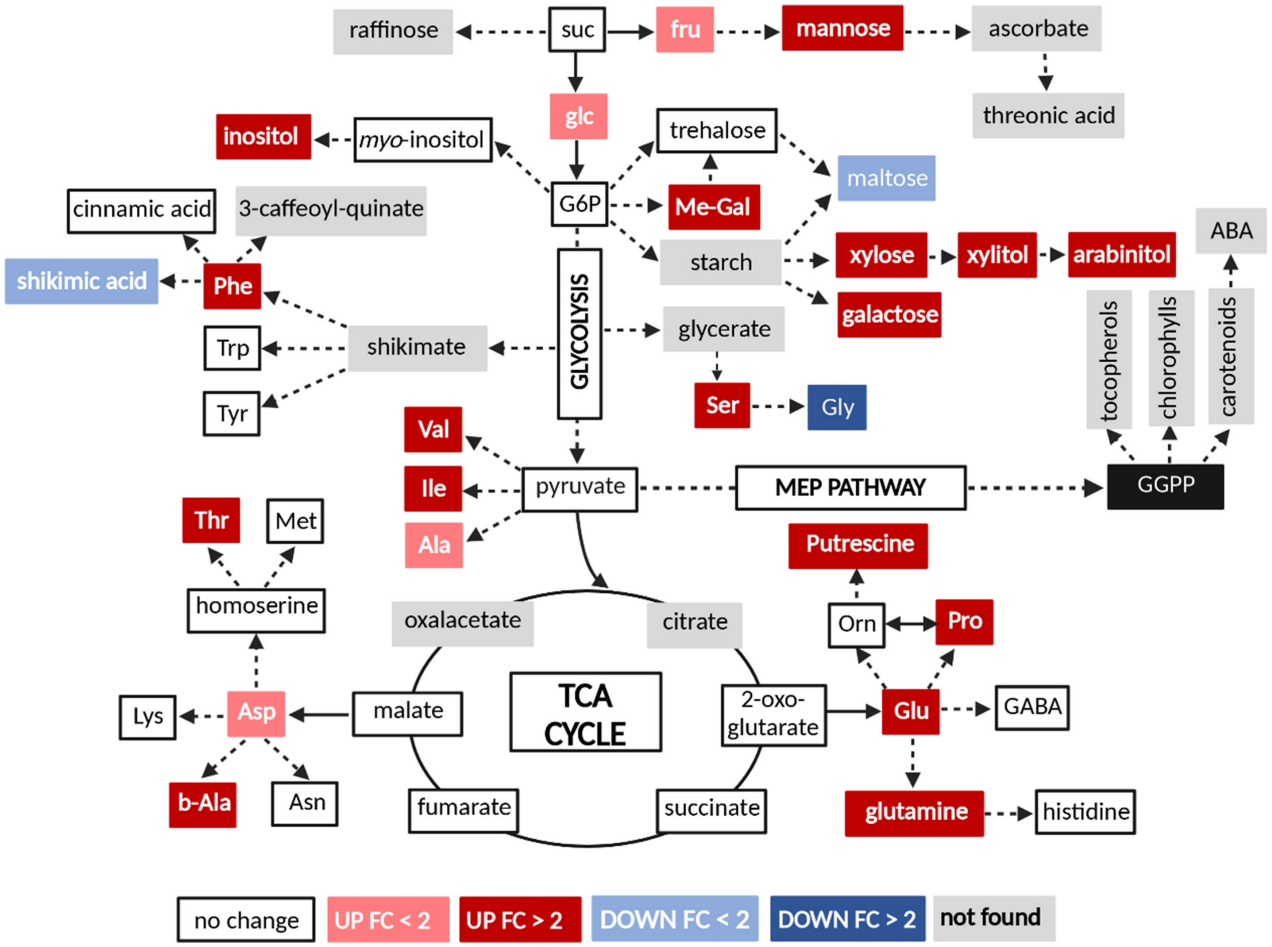
Changes in the levels of selected differentially concentrated metabolites of susceptible cultivar Yunyan87 during *R. pseudosolanacearum* infection. Colors represent statistically significant fold-change (FC) values (*t* test, p < 0.05) of metabolite levels in xylem sap from infected tobacco plants relative to those in healthy plants controls. Suc, sucrose; Glc, glucose; Fru, fructose; Me-Gal, methyl-alpha-D-glucopyranoside; Ser, serine; Gly, glycine; Phe, phenylalanine; Trp, tryptophan; Tyr, tyrosine; Val, valine; Ile, isoleucine; Ala, alanine; Glu, glutamic acid; Orn, ornithine; Pro, proline; Asp, aspartic acid; Thr, threonine; Met, methionine; Lys, lysine; b-Ala, beta-alanine; Asn, asparagine. This figure was created by bio Render (https://biorender.com/).

Exploration of certain metabolites altered in the susceptible cultivar indicated a dramatic increase in methyl-alpha-D-glucopyranoside and arabinitol (Figure 6). These two compounds could be originated from glucose and xylose, respectively. Furthermore, infected sap also displayed increased levels of amino acids derived from glycerate (Ser), shikimate (Phe), pyruvate (Val, Ile and Ala), 2-oxoglutarate (Glu and Pro) and malate (Asp, Thr, and beta-alanine). Derivatives from glutamic acid, glutamine, and putrescine were also enriched in infected plants. Alterations in the sugar metabolism also included accumulation of xylose, xylitol, galactose, inositol, fructose, mannose, and glucose. Finally, decreased levels of maltose, glycine, and shikimic acid in infected xylem sap were also observed (Figure 6). It is known that cell wall degrading enzymes of *R. solanacearum* could release cellulose-derived metabolites like cellobiose and gentiobiose during infection (Genin and Denny, 2012). In addition, it has been proposed that *R. solanacearum* may use type III secretion system to manipulate the host to release nutrients into the xylem (Deslandes and Genin, 2014). RipI promotes the biochemical activation of glutamate decarboxylases (GADs) in plant cells, enhancing the production of gamma-aminobutyric acid (GABA) to support *R. solanacearum* nutrition during plant infection (Xian et al., 2020). However, most of metabolites were not enhanced during the interaction of this pathogen with resistant tobacco cultivar K326 (Supplementary Table S2).

### Insights Into Pathogen Preferred Carbon Sources in Tobacco Plant

The fast proliferation of *R. solanacearum* in xylem sap should be sustained by nutrients that the pathogen takes up in this environment (Gerlin et al., 2021). The amino acids, organic acids, sugars, and other metabolites enriched in xylem sap might favor *R. pseudosolanacearum* growth *in planta*. Enriched amino acids included aspartic acid, beta-alanine, glutamine, isoleucine, leucine, phenylalanine, proline, serine, threonine, and valine (Figures 3A, 4A). Glutamine, asparagine, and gamma aminobutyric acid were identified as the major organic components of xylem sap in tomato (Zuluaga et al., 2013). Together with leucine, isoleucine and histidine decreased under *R. solanacearum* inoculation *in vitro* (Zuluaga et al., 2013; Gerlin et al., 2021). However, we did not detect any of these compounds decreasing in the tobacco xylem after infection and most of them even increased significantly. This correlates with previous findings in which glutamic acid, phenylalanine, beta-alanine, leucine, and glycine were enriched in xylem sap during *R. solanacearum* infection (Lowe-Power et al., 2018a; Zeiss et al., 2019). We thus hypothesize that *R. pseudosolanacearum* causes a deep plant metabolic reprogramming during infection to favor the production of compounds that can sustain its growth. In support of this theory is the recent finding that *R. solanacearum* effector protein RipI interacts with plant glutamate decarboxylases to alter plant metabolism, enhancing GABA production to support bacterial growth (Xian et al., 2020).

### Metabolic Signatures of *Solanaceae* Plants in Response to Bacterial Wilt

In this study, we found that beta-alanine, phenylalanine, leucine, glycine, galactonic acid, 3-hydroxybutyric acid, shikimic acid, ribose, and putrescine were both enriched in tomato and tobacco plants in response to *R. pseudosolanacearum* infection, putrescine being the most enriched in both plant xylems (Supplementary Table S2). Putrescine cannot be used as a carbon source or nutrient by *R. solanacearum* and is copiously produced by this bacterium. Thus, this molecule would be mostly derived from the pathogen and it would act as a virulence metabolite by inducing the wilting symptoms (Lowe-Power et al., 2018a; Gerlin et al., 2021). A recent study showed that putrescine could be produced by tomato cells hijacked by a *R. solanacearum* effector (Wu et al., 2019).

Certain studies have demonstrated that 3-hydroxybutyric acid was identified as a storage compound in *R. solanacearum* and phylogenetically close β-proteobacteria, precursor of polyhydroxybutyrate (Terpolilli et al., 2016; Gerlin et al., 2021). As previously reported, 3-hydroxybutyric acid increased in the xylem sap of *R. solanacearum* infected plants, and this metabolite could be excreted by *R. solanacearum*, and consumed before returning to the soil (Lowe-Power et al., 2018a; Gerlin et al., 2021). This is in agreement with our finding that 3-hydroxybutyric acid was enriched in the xylem of infected tobacco plant. However, whether this metabolite produced by plants or bacteria is still unclear, as the biosynthetic pathway of the metabolite is present in both organisms (Yuan et al., 2016).

### Certain Metabolic Pathways Are Involved in Plant Defense in Response to *R. pseudosolanacearum* Attack

Several amino acid pathways contribute to defense responses of plants exposed to infection by *R. solanacearum*. Proteomic and transcriptomic analysis revealed that the methionine cycle (MTC) and γ-aminobutyric acid (GABA) play a key role in plant defense against *R. solanacearum*. Silencing of MTC-associated genes *SAHH1* and *MS1* and GABA biosynthesis gene *GAD2* in tomato leads to decreased resistance against *R. solanacearum* (Wang et al., 2019). Certain studies have proven that GABA levels were rapidly increased in plants in response to various biotic stresses (Park et al., 2010; Ramesh et al., 2017). Arabidopsis wat1 (walls are thin1)-mediated resistance to *R. solanacearum* is mediated by cross-regulation of salicylic acid and tryptophan metabolism (Denance et al., 2013). Plant metabolic pathways mediated by pyruvate decarboxylases (PDCs) also contribute to plant tolerance to bacterial wilt. And an effector protein secreted by *R. solanacearum*, RipAK, interacts with PDCs and is involved in plant resistance to biotic and abiotic stresses (Wang et al., 2021). Moreover, application of pyruvic acid and acetic acid (substrate and product of the PDC pathway) enhanced plant tolerance to bacterial wilt. In this study, we found that certain amino acids such as alanine, phenylalanine, leucine, and glycine were enriched in tobacco and tomato after *R. pseudosolanacearum* infection (Figure 6; Supplementary Table S2). The role of these amino acids in plant defense against bacterial wilt needs further investigation.

## Conclusion

In conclusion, GC-MS-based metabolomic analysis revealed relatively metabolic profiling in two tobacco cultivars that are quantitatively resistant (K326) or susceptible (Yunyan87) to initial infection by *R. pseudosolanacearum*. A total of 48 different concentrated metabolites were identified in tobacco cultivar Yunyan87. The contents of metabolites related to amino acid metabolism, sugar metabolism, and organic acid metabolism were enriched in infected tobacco xylem sap. Certain amino acids such as alanine, phenylalanine, leucine, and glycine were enriched in tobacco and tomato after *R. pseudosolanacearum* infection. The role of certain amino acids needs further and more detailed investigation.

## Data Availability Statement

The datasets presented in this study can be found in online repositories. The names of the repository/repositories and accession number(s) can be found in the article/**Supplementary Material**.

## Author Contributions

WD and LY conceived and designed the experiments. LY and MV performed the experiments. LY, ZW, and MV analyzed the data. LY, WD, and MV wrote and revised the paper. All authors contributed to the article and approved the submitted version.

## Funding

The research was supported by the key project of the China National Tobacco Corporation (110201901042), the National Natural Science Foundation of China (31972288), the Chongqing Special Postdoctoral Science Foundation, PID2019-108595RB-I00/AEI/10.13039/501100011033 and CEX2019-000902-S from the Spanish Ministry of Science and Innovation, and the CERCA Program from the Catalan Government (Generalitat de Catalunya) and China Postdoctoral Science Foundation (2021M702707).

## Conflict of Interest

The authors declare that the research was conducted in the absence of any commercial or financial relationships that could be construed as a potential conflict of interest.

## Publisher’s Note

All claims expressed in this article are solely those of the authors and do not necessarily represent those of their affiliated organizations, or those of the publisher, the editors and the reviewers. Any product that may be evaluated in this article, or claim that may be made by its manufacturer, is not guaranteed or endorsed by the publisher.

## Supplementary Material

Supplementary Figure S1 Metabolome analysis of tobacco cultivar in response to *R. pseudosolanacearum* infection. **(A)** Tobacco plants were soil-soak inoculated with *R. pseudosolanacearum* CQPS-1, after inoculation for 7 days, xylem sap was harvested from infected and healthy plants. **(B)** PCA analysis of metabolites change of infected tobacco plant and healthy plant. SI means susceptible tobacco cultivar infected with *R. pseudosolanacearum*, SNI indicates susceptible cultivar without pathogen infection. RI means moderately resistant tobacco cultivar K326 inoculated with *R. pseudosolanacearum*, RNI means K326 tobacco plants without inoculated with *R. pseudosolanacearum*. **(C)** The clustering tree of changed metabolites of tobacco xylem sap under *R. pseudosolanacearum* infection.

Supplementary Figure S2 Different effect of tobacco metabolome of two tobacco cultivars in response to *R. pseudosolanacearum* infection. **(A)** PCA analysis of metabolite changes in infected and healthy tobacco plants. SI means susceptible tobacco cultivar infected with *R. pseudosolanacearum*, SNI indicates susceptible cultivar without pathogen infection. **(B)** The clustering tree of changed metabolites of tobacco Yunyan87 xylem sap under *R. pseudosolanacearum* infection. **(C)** PCA analysis of metabolites change of infected and healthy tobacco plants. RI means moderately resistant tobacco cultivar infected with *R. pseudosolanacearum*, RNI indicates moderately resistant cultivar without pathogen infection. **(D)** The clustering tree of changed metabolites in tobacco K326 xylem sap during *R. pseudosolanacearum* infection.

Supplementary Table S1 Relative quantification of metabolites in tobacco cultivars xylem sap.

Supplementary Table S2 Xylem sap metabolites altered by bacterial wilt disease in tobacco and tomato plants.
