# Supplementary figures and images for: Metabolic Profiling of Resistant and Susceptible Tobaccos Response Incited by Ralstonia pseudosolanacearum Causing Bacterial Wilt
Source: Front Plant Sci. 2022 Jan 7;12:780429. doi: 10.3389/fpls.2021.780429 (PMC8780990; doi:10.3389/fpls.2021.780429)

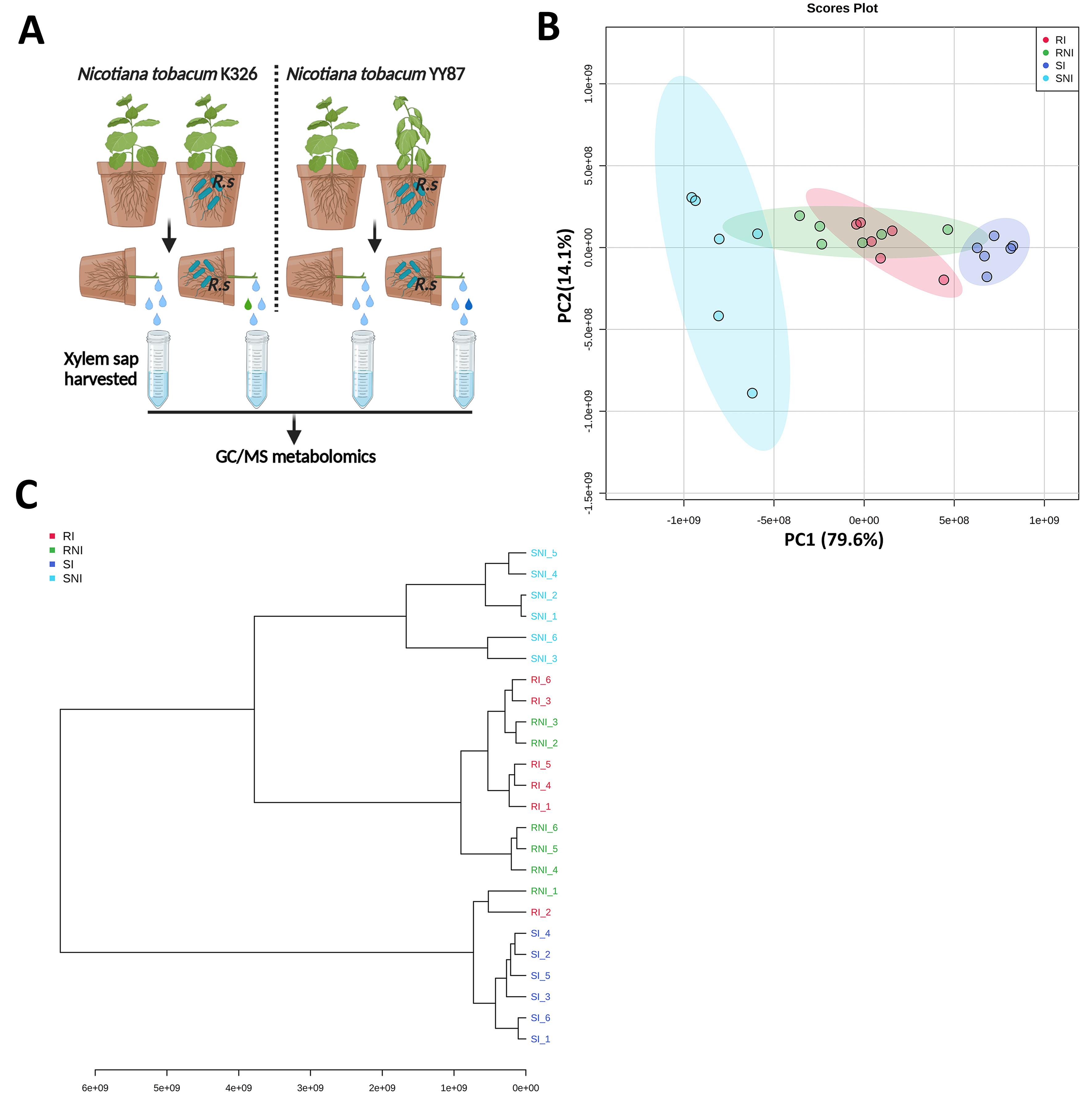

Supplement: Supplementary Figure S1 — Metabolome analysis of tobacco cultivar in response to R. pseudosolanacearum infection. (A) Tobacco plants were soil-soak inoculated with R. pseudosolanacearum CQPS-1, after inoculation for 7 days, xylem sap was harvested from infected and healthy plants. (B) PCA analysis of metabolites change of infected tobacco plant and healthy plant. SI means susceptible tobacco cultivar infected with R. pseudosolanacearum, SNI indicates susceptible cultivar without pathogen infection. RI means moderately resistant tobacco cultivar K326 inoculated with R. pseudosolanacearum, RNI means K326 tobacco plants without inoculated with R. pseudosolanacearum. (C) The clustering tree of changed metabolites of tobacco xylem sap under R. pseudosolanacearum infection. [file Image_1.tif]

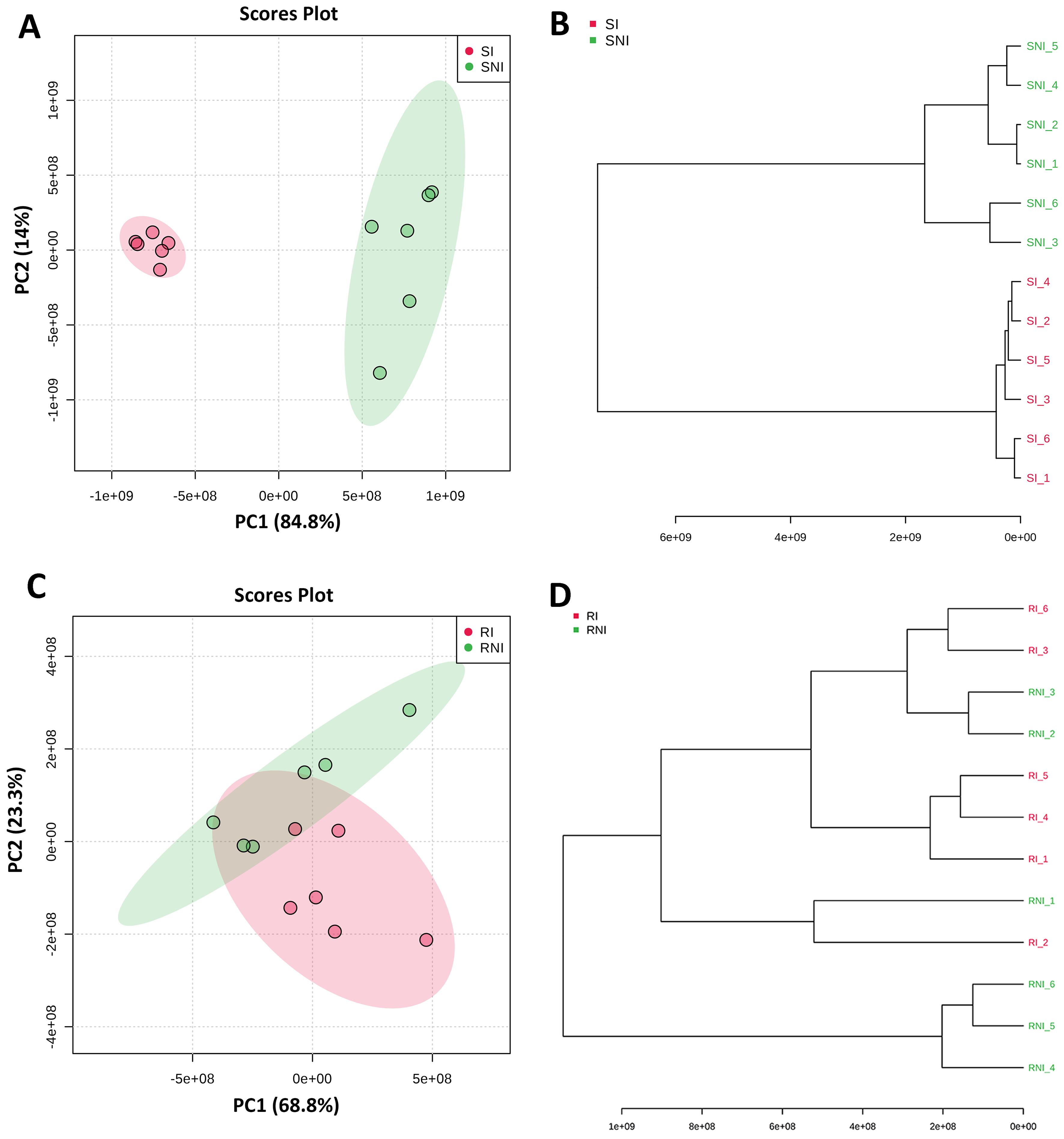

Supplement: Supplementary Figure S2 — Different effect of tobacco metabolome of two tobacco cultivars in response to R. pseudosolanacearum infection. (A) PCA analysis of metabolite changes in infected and healthy tobacco plants. SI means susceptible tobacco cultivar infected with R. pseudosolanacearum, SNI indicates susceptible cultivar without pathogen infection. (B) The clustering tree of changed metabolites of tobacco Yunyan87 xylem sap under R. pseudosolanacearum infection. (C) PCA analysis of metabolites change of infected and healthy tobacco plants. RI means moderately resistant tobacco cultivar infected with R. pseudosolanacearum, RNI indicates moderately resistant cultivar without pathogen infection. (D) The clustering tree of changed metabolites in tobacco K326 xylem sap during R. pseudosolanacearum infection. [file Image_2.tif]
